# Restorative effects of gallic acid against sub-chronic hepatic toxicity of co-exposure to zinc oxide nanoparticles and arsenic trioxide in male rats

**DOI:** 10.1016/j.heliyon.2023.e17326

**Published:** 2023-06-15

**Authors:** Khaled Abo-EL-Sooud, Yasmina M. Abd-El Hakim, Mohamed M.M. Hashem, Abeer E. El-Metwally, Bayan A. Hassan, Hayat H.M. El-Nour

**Affiliations:** a Department of Pharmacology, Faculty of Veterinary Medicine, Cairo University, Giza 12613, Egypt; b Department of Forensic Medicine and Toxicology, Faculty of Veterinary Medicine, Zagazig University, Zagazig 44519, Egypt; c Pathology Department, Animal Reproduction Research Institute, Giza 3514805, Egypt; d Pharmacology Department, Faculty of Pharmacy, Future University, Cairo 11835, Egypt; e Biology of Reproduction Department, Animal Reproduction Research Institute, Giza 3514805, Egypt

**Keywords:** Zinc oxide nanoparticles, Arsenic trioxide, Gallic acid, Bilirubin, Bcl-2, GPx, MDA, Bax

## Abstract

**Background and objectives:**

This study aimed to assess the effect of zinc oxide nanoparticles (ZNPs) and/or arsenic trioxide (ATO) exposure on the liver of adult male Sprague Dawley rats. Moreover, the probable ameliorative impact of gallic acid (GA) against ZNPs and ATO-induced hepatotoxicity and the possible underlying mechanisms were evaluated.

**Methods:**

Sixty male Sprague Dawley rats were distributed into six groups. The 1^st^ and 2^nd^ groups were orally given distilled water (1 ml/kg) and 20 mg GA/kg b. wt, respectively. The 3^rd^ and 4^th^ groups were orally given 100 mg ZNPs/kg b. wt and 8 mg ATO/kg b. wt, respectively. The 5^th^ group was co-administered ZNPs and ATO at the doses mentioned above. The last one was co-administered ZNPs, ATO, and GA at the earlier described doses. All tested compounds were orally given once a day for 60 successive days. Then, serum levels of alkaline phosphatase (ALP), alanine aminotransferase (ALT), aspartate aminotransferase (AST), total, direct, indirect bilirubin, triglycerides, total cholesterol, HDL, VLDL, and LDL were estimated. The hepatic content of malondialdehyde (MDA), superoxide dismutase (SOD), and glutathione peroxidase (GPx) was evaluated. Moreover, Bcl-2 and Bax's reactive proteins were immunohistochemically detected, and Zn and As residual patterns in hepatic tissues were assessed.

**Results:**

ZNPs, ATO, and ZNPs+ATO-exposed rats showed significantly (*P* < 0.001) elevated serum AST (219%, 233%, and 333%), ALT (300%, 400%, and 475%), ALP (169%, 205%, and 294%), and total bilirubin (42%, 68%, and 109%) compared to the control ones. On the other hand, a significantly (*P* < 0.001) declined SOD (58%, 49%, and 43%) and GPx (70%, 63%, and 56%) but increased MDA (133%, 150%, and 224%) was recorded in the hepatic tissues of ZNPs, ATO, and ZNPs+ATO exposed rats, respectively, relative to the control rats. Moreover, the hepatic tissues of the ZNPs, ATO, and ZNPs+ATO exposed rats showed a significant (*P* < 0.001) decrease in Bcl-2 (28%, 33%, and 23%) but elevation in Bax (217%, 267%, and 236%) immunoreactivities compared to the control rats. These findings were consistent with the microscopic alterations in the hepatic architecture and accumulation of Zn and As. Furthermore, a notable hyperlipidemic condition was recorded following ZNPs and/or ATO exposure. On the contrary, GA notably reduced hepatic enzymes compared to ZNPs+ATO-exposed rats. Additionally, GA markedly improved ZNPs+ATO-afforded liver tissue damage and apoptotic events.

**Conclusion:**

Overall, GA oral dosing significantly mitigated the negative effects of ZNPs and ATO on the liver by improving the antioxidant defense system and controlling apoptotic changes.

## 1 Introduction

Zinc oxide nanoparticles (ZNPs) are one of the most highly marketable NPs in the world [1,2]. While using ZNPs for medicinal purposes is expanding, the biochemical modulation of living cell functions remains deficient [3,4]. Therefore, the potential toxicity of ZNPs is an imperative issue as they have been used in various commercial products such as paints, toothpaste, cosmetics, and photographic materials, and as a food additive [[5], [6], [7]]. Moreover, these nanoparticles can effectively treat arthritic gout [8]. ZNPs cause cytotoxicity by the induction of oxidative stress via Zn ions [9]. ZNPs at over 50 mg/kg concentration significantly altered rats' hepatic enzymes and induced oxidative stress [10].

The potential antineoplastic efficacy of arsenic trioxide (ATO) against acute promyelocytic leukemia makes it the top agent in several countries [11]. ATO can inhibit the proliferation and initiate Caspase-mediated apoptosis in tumor cells [12]. Moreover, it inactivates the SH-containing proteins and alters the mitochondrial roles [13]. Nevertheless, its use is guarded by its untoward effects, especially on vital organ functions [14]. Consumption of arsenic (As)-contaminated drinking water constitutes an additional major global health concern [15].

Daily administration of 3 mg ATO/kg b. wt in Wistar rats induces lipid peroxidation and oxidative stress in the liver and kidney [16,17]. Arsenic causes oxidative stress through cellular macromolecule damage, including DNA, protein, and lipids, with subsequent high production levels of free oxidative radicals [18]. Additionally, ATO intragastric infusion at 10 mg/kg b. wt. Produced monomethyl arsenic acid, dimethylarsinic acid, and trimethylarsine oxide in rats' serum, which caused excessive reactive oxygen species (ROS) regeneration, cell apoptosis, and acute hepatic damage [19]. Exposure to ATO increased the activity of caspase-3 in the hepatic cells [20].

Different sources of environmental pollutants co-exist concurrently with NPs, especially heavy metals that are extensively distributed [[21], [22], [23]]. There are various co-exposure scenarios for ZNPs and As in the environment and in the medical and industrial fields. For example, ZNPs and As could co-exist in the soil. This is due to the accumulation of As in soils due to anthropogenic activities like herbicide over-use, coal combustion, and timber preservation [24] and the wide application of ZNPs as fertilizer [25,26]. Additionally, ZNPs has widely used in many industrial and medicinal products such as rubber, coating, paint, and cosmetics [27]. In parallel, As has been detected in many of the earlier products [28,29]. Hence, animals and humans are at high risk of joint exposure to ZNPs and As.

Gallic acid (GA) has a unique phenolic moiety with potent anti-inflammatory, antioxidant, and anticancer activities [30]. GA significantly decreases serum hepatic enzymes, normalizes the expression of pro-inflammatory cytokines, interleukins, cyclooxygenase 2 (COX2) [31], and tumor necrosis factor-alpha (TNF-α) [32], and upregulates antioxidant gene expression of SOD and CAT [33] with modulation of antioxidant/pro-oxidant balance and inhibits ROS production [34]. GA's possible protective effect against subchronic ZNP+ATO toxicity is still unknown. Hence, this study examined the effects of separate or joint ZNPs and ATO exposure on the liver and GA's hepatoprotective actions at their co-exposure in male rats.

## 2 Materials and methods

### 2.1 Chemicals and reagents

Zinc oxide nanoparticles ZNPs, molecular weight (MW) = 81.39; average nanosize 30 ± nm particle size), gallic acid (C_7_H_6_O_5._ H_2_O, MW = 188.14), and arsenic trioxide (ATO).2H_2_O, MW = 197.84, 99% purity) were obtained from Alpha Chemica (Mumbai, India). All additional reagents/chemicals were purchased from Sigma Company and were of analytical quality (St. Louis, MO).

### 2.2 ZNPs suspension preparation

The ZNPs particles were dispersed in distilled water (10 mg/ml). Following the protocol of Ramadan et al. [35], a fresh suspension was prepared daily, and the suspensions were sonicated for 15 min with an ultrasonic cleaner (500 W, 42 kHz, 25 °C, FRQ-1010 H T, Hangzhou, China). The prepared ZNPs suspension was stirred on a vortex agitator directly before animal administration. The earlier method has achieved high Zn dispersion stability [36].

### 2.3 Animals and experimental design

Adult male Sprague Dawley rats (n = 60) were bought from the National Research Center's breeding section (Giza, Egypt). All rats were kept in well-ventilated, clean steel mesh cages with a 12-h light-dark cycle at 21–24 °C and 50–60% relative humidity. To keep the cages dry, wood-shaving bedding was used. Rats had unlimited access to tap water, and standard rodent food throughout the experiment. Before testing, rats were given a two-week acclimatization period prior to the commencement of the experiment. The experimental protocol was authorized by Cairo University's research committee on the ethics of animal use, with the reference number VET CU 2009 2022461 which followed the general criteria of the National Institutes of Health Guide for the Care and Use of Laboratory Animals in Scientific Investigations. Rats were weighed and assigned to six groups at random (n = 10 each):

**G1: Control group:** each rat received 1 ml/day of distilled water.

**G2: Gallic acid (GA):** orally administered 20 mg/kg b. wt. GA dissolved in distilled water according to the dose used in the previous study [37].

**G3: Zinc oxide nanoparticles (**ZNPs**):** orally administered 100 mg ZNPs/kg b. wt. According to the dose used in the earlier study [38], ZNPs dissolved in distilled water were dispersed by ultrasonic vibration for 20 min.

**G4: Arsenic trioxide (ATO):** orally administered 8 mg ATO/kg b. wt. Dissolved in distilled water according to the dose used in the previous study [39].

**G5: ZNPs**+**ATO:** co-administered ZNPs and ATO at the same previous routes and doses separately with 5 min between.

**G6: ZNPs**+**ATO**+**GA:** co-administered ZNPs and ATO separately with 5 min between, followed by GA after 1 h at the same previous routes and doses.

All treatments were given orally via orogastric gavage once a day between 8 a.m. and 10 a.m. using a feeding needle for 60 days (16 gauge). The dosage volumes were adjusted weekly according to the recorded average weekly weight of the rats.

Every week, all treatments were re-adjusted depending on the rats' body weight changes. Pain, discomfort, damage, abnormal behavior, distress, mucous membrane color, breathing patterns, morbidity, and mortality were closely monitored during the experimental period. The consumed amount of food and the weight of the animals were measured weekly.

### 2.4 Sampling

All rats in each group were fasted overnight after the last dose to allow accurate measurement of body and organ weights and reliable estimation of the biochemical indicators [40]. Then, the rats were weighed, anesthetized, and euthanized by cervical dislocation. Jugular vein blood samples were collected into a plain tube, allowed to clot at room temperature for 20 min, centrifuged for 10 min at 664×*g*, and the resulting serum was stored at −20 °C for later biochemical analysis. The livers were collected, washed with physiological saline, and weighed. The liver specimens were divided into three sets. The first set was fixed in a 10% buffered neutral formalin solution for histopathological and immunohistochemical investigation. The second one was used to prepare tissue homogenates for the assays defined below. The last one was kept at 4 °C till analysis of metal residues.

### 2.5 Estimation of hepatic enzymes and bilirubin levels

Commercial Biodiagnostic kits (Giza, Egypt) were used to estimate serum alkaline phosphatase (ALP), alanine aminotransferase (ALT), and aspartate aminotransferase (AST) activities following the methods of Reitman and Frankel [41] and Kind and King [42], respectively. The total and direct serum bilirubin was assessed based on the procedures of Walters and Gerarde [43] method.

### 2.6 Lipid profile assay

Serum cholesterol was assessed based on the Abel et al. [44] protocol. The triglycerides were evaluated consistently with the method of Bucolo and David [45]. The serum high–density lipoprotein cholesterol (HDL-C) concentration was estimated according to Assmann et al. [46].

The low–density lipoprotein cholesterol (LDL-C) and the very low-density lipoproteins (VLDL) values were estimated by the formula of Friedewald et al. [47] as follows: VLDL-C = TG/5 and LDL-C (mg/dL) = TC − (HDL-C + VLDL-C).

### 2.7 Oxidative stress and antioxidant indicators in liver homogenate

The liver homogenate was prepared following Kaplan and Utiger [48] method. A liver portion was homogenized in phosphate buffer saline (0.1 M PBS with pH 7.4). The homogenates were then centrifuged for 30 min at 4 °C at 10,000 rpm, and the supernatants were kept at 70 °C until oxidative stress parameters analysis. The hepatic tissue content of MDA, SOD, and GPx was estimated in liver homogenate based on the colorimetric method using Biodiagnostic kits, diagnostic and research reagents, Egypt following the procedures formerly described protocols [[49], [50], [51]].

### 2.8 Histopathological examination

Each animal's liver specimens were dissected and fixed in a 10% formalin. Then, they were dehydrated with ascending alcohol concentrations, cleared in xylene, embedded, and blocked in paraffin. 4 μm sections were taken, stained with Hematoxylin and Eosin (H & E), and proceeded for the routine protocol of Bancroft and Layton [52]. Samples of all rats were examined randomly with a light microscope (Olympus, Tokyo, Japan) at different magnifications and analyzed to find histological alterations.

### 2.9 Histochemical examination

Periodic Acid Schiff's stain (PAS) was performed to detect the glycogen in the cytoplasm of the hepatocytes, according to Layton et al. [53]**.**

### 2.10 Immunohistochemical analysis

According to Pedrycz and Czerny [54] and Kandemir et al. [55], 4 μm cross-sections taken from each paraffin block were placed on slides coated with poly-l-lysine to determine Bcl-2 and Bax immunohistochemical changes.

### 2.11 Determination of Zn and as hepatic contents

Each liver sample was microwave-digested with 8 ml of nitric acid and 1 ml of 30% hydrogen peroxide. Then, the Zn and As contents were determined by a coupled plasma-Optical Emission Spectrometer (ICP-OES, model 5100, Agilent, Santa Clara, CA) with Synchronous Vertical Dual View (SVDV). The standard intensity curve was settled for each set of samples using a blank and three or more Merck Company standards (Germany). Reference standards from Merck were used to verify the accuracy and precision of the metal's measurements. The results were validated using known concentrations of trace elements from the National Institute of Standards and Technology (NIST).

### 2.12 Statistical analysis

The data were analyzed with SPSS version 14 and one-way analysis of variance (ANOVA) (SPSS, Chicago, IL, USA). To compare means, Tukey's Multiple Range Test was used. The differences between groups were considered significant at p-value <0.05, <0.01, and <0.001. A Shapiro-Wilk W test was used to ensure all data were distributed normally.

## 3 Results

### 3.1 Effects on body weight change

No mortalities were recorded throughout the experiment. The ZNPs, ATO, and ZNPs+ATO-exposed rats showed a significant (*P* < 0.001) reduction in the final body weight (84%, 81%, and 78%, respectively) and body weight gain (32%, 16%, and 14%, respectively) relative to the control group (Table 1). On the other hand, GA+ZNPs+ATO-treated rats displayed a significant (*P* < 0.001) increase in the final body weight and body weight gain to be 115% and 432%, respectively, relative to the ZNPs+ATO-co-exposed ones.

**Table 1:** Effect of gallic acid (GA) oral dosing on body weight change and hepatosomatic index of Sprague Dawley rats exposed to zinc dioxide nanoparticles (ZNPs) and/or arsenic trioxide (ATO) for 60 days.

Estimated parameters	Control	GA	ZNPs	% of control*	ATO	% of control*	ZNPs+ATO	% of control*	GA+ZNPs+ATO	% of restoration^≠^
Initial Body weight (g)	213.33 ± 4.71	206.67 ± 4.71	206.67 ± 4.71	97	206.67 ± 4.71	97	200.00 ± 0.00	94	206.67 ± 4.71	103
Final body weight (g)	266.00^a^ ±5.72	272.67^a^±2.62	223.67^c^±0.24	84	215.00^cd^ ± 7.18	81	207.33 ^d^ ± 0.62	78	238.33 ^b^ ± 3.06	115
Body weight gain (g)	52.67 ^b^ ± 5.91	66.00^a^±6.16	17.00 ^d^ ± 4.60	32	8.33 ^d^ ± 2.66	16	7.33 ^d^ ± 0.62	14	31.67^c^±1.65	432
Liver weight (g)	6.46 ± 0.27	6.98 ± 0.14	6.13 ± 0.43	95	6.35 ± 0.41	98	6.45 ± 0.10	100	6.00 ± 0.32	93
Hepatosomatic index (%)	2.43^c^ ±0.12	2.56 ^bc^±0.03	2.74 ^abc^±0.19	113	2.96 ^ab^ ± 0.19	122	3.11^a^±0.06	128	2.52^c^±0.13	81

*% of control = (mean value of the intoxicated group/mean values of the control group)*100. ^≠^ % of restoration= (mean value of GA+ZNPs+ATO group/mean values of ZNPs+ATO group)*100. Means within the same row carrying different superscripts (a, b, c, and d) are significantly different at *p* < 0.05. The values shown are means ± SE. n = 10.

No significant change was recorded in the hepatosomatic index of the ZNPs-exposed rats compared to the control group (Table 1). Nevertheless, the ATO and ZNPs+ATO-exposed rats displayed a significant (*P* = 0.01) increase in the hepatosomatic index to 122% and 128%, respectively, compared to the control ones. On the contrary, GA+ZNPs+ATO-treated rats demonstrated a significant (*P* = 0.01) reduction in the hepatosomatic index to be 81% relative to the ZNPs+ATO-co-exposed ones.

### 3.2 Effects on liver function indicators

The serum hepatic enzymes activities, including ALP, ALT, and AST, were significantly (*P* < 0.001) increased in the ZNPs (169%, 300%, and 219% respectively), ATO (205%, 400%, and 233%, respectively), and ZNPs+ATO (294%, 475%, and 339% respectively)-exposed rats compared to the control group (Table 2). Besides, a significant (*P* < 0.001) elevation in the total and direct bilirubin was recorded in the ZNPs (142% and 148%, respectively), ATO (168% and 158%, respectively), and ZNPs+ATO (209% and 237% respectively)-exposed group compared to the control group.

**Table 2:** Effect of gallic acid (GA) oral dosing on serum levels of biochemical parameters of rats exposed to zinc dioxide nanoparticles (ZNPs) and/or arsenic trioxide (ATO) for 60 days.

Estimated parameters	Control	GA	ZNPs	% of control*	ATO	% of control*	ZNPs+ATO	% of control*	GA+ZNPs+ATO	% of restoration^≠^
AST (U/dl)	7.00^c^±1.22	5.67^c^±0.47	15.33 ^b^ ± 1.93	219	16.33 ^b^ ± 2.25	233	23.33^a^±2.01	333	15.33 ^b^ ± 0.24	66
ALT (U/m)	4.00 ^cd^ ± 0.41	2.67 ^d^ ± 0.24	12.00 ^b^ ± 0.41	300	16.00^a^±2.12	400	19.00^a^±1.47	475	7.00^c^±0.71	37
ALP (U/dl)	36.67 ^d^ ± 2.62	36.00 ^d^ ± 1.08	62.00^c^±2.55	169	75.33 ^b^ ± 1.55	205	107.67^a^±6.11	294	66.33 ^bc^±0.85	62
Total bilirubin (mg/dl)	0.64^c^±0.07	0.40^c^±0.04	0.91 ^b^ ± 0.07	142	1.08 ^b^ ± 0.12	168	1.34^a^±0.13	209	0.97 ^b^ ± 0.06	72
Direct bilirubin (mg/dl)	1.30 ^d^ ± 0.03	1.05^e^±0.05	1.92^c^±0.05	148	2.05^c^±0.09	158	3.08^a^±0.01	237	2.53 ^b^ ± 0.10	82
Total cholesterol (mg/dl)	180.33^c^±2.59	160.00 ^d^ ± 1.08	201.67^b^ ± 4.13	112	229.00^a^±4.71	127	239.33^a^±5.91	133	187.00^c^±7.49	78
Triglyceride (mg/dl)	77.33 ^d^ ± 1.43	59.00^e^±2.68	89.33^c^±2.25	116	110.00 ^b^ ± 2.12	142	123.00^a^±1.22	159	95.67^c^±2.78	78
LDL (mg/dl)	25.67 ^cd^ ± 0.85	23.67 ^d^ ± 1.31	38.33 ^ab^ ± 3.30	149	43.22 ^b^ ± 2.61	168	45.33^a^±2.09	177	31.67 ^bc^±2.49	70
VLDL (mg/dl)	16.13 ^de^ ± 1.02	13.93^e^±0.66	19.07 ^cd^ ± 0.87	118	34.60 ^b^ ± 2.11	215	42.47^a^±2.08	263	22.00^c^±1.69	52
HDL (mg/dl)	23.18 ^b^ ± 0.91	35.41^a^±1.06	18.43^c^±0.13	219	13.11 ^d^ ± 0.77	57	11.11 ^d^ ± 1.11	48	21.66 ^b^ ± 0.67	195

*% of control = (mean value of the intoxicated group/mean values of the control group)*100. ^≠^ % of restoration= (mean value of GA+ZNPs+ATO group/mean values of ZNPs+ATO group)*100. AST: Aspartate aminotransferase; ALT: Alanine aminotransferase; ALP: alkaline phosphatase; LDL: low-density lipoprotein; VLDL: Very low-density lipoprotein; HD: High-density lipoprotein. Means within the same row carrying different superscripts (a, b, c, and d) are significantly different at *p* < 0.05. The values shown are means ± SE. n = 10 group.

On the other hand, GA treatment significantly suppressed the ZNPs+ATO-induced increment of ALP, ALT, AST, total, and direct bilirubin to be 62%, 37%, 66%, 72%, and 82%, respectively, compared to the ZNPs+ATO-co-exposed group.

### 3.3 Effects on lipid profile

Differences in the serum lipid profile of rats orally exposed to ZNPs/or ATO for 60 days and those orally treated with GA are displayed in Table 2. Compared with the control group, the serum levels of TC, TG, LDL, and VLDL were significantly (*P* < 0.001) increased in the ZNPs (112%, 116%, 149%, and 118%, respectively), ATO (127%, 142%, 168%, and 215% respectively), and (133%, 159%, 177%, and 263% respectively)-exposed group. On the contrary, a significant (*P* < 0.001) decline in the HDL level was evident in rats exposed to ZNPs, ATO, and ZNPs+ATO to be 80%, 57%, and 48%, respectively, relative to the control group. Of note, the ZNPs and ATO co-exposed rats showed a significantly (*P* < 0.001) higher TG and VLDL than those exposed to each individually. In contrast, GA+ZNPs+ATO co-treated group showed a significantly (*P* < 0.001) lower TC, TG, LDL, and VLDL at 78%, 78%, 70%, and 52%, respectively, compared to the ZNPs+ATO exposed group. Besides, a significantly (*P* < 0.001) higher HDL was evident in the GA+ZNPs+ATO co-treated rats to be 195% compared to the ZNPs+ATO exposed ones. Noteworthy, GA+ZNPs+ATO-treated rats showed marked improvement in TC, LDL, and HDL, where no significant changes exist compared to the control rats.

### 3.4 Effects on hepatic oxidative stress and lipid peroxidation indicators

**Fig. 1:**
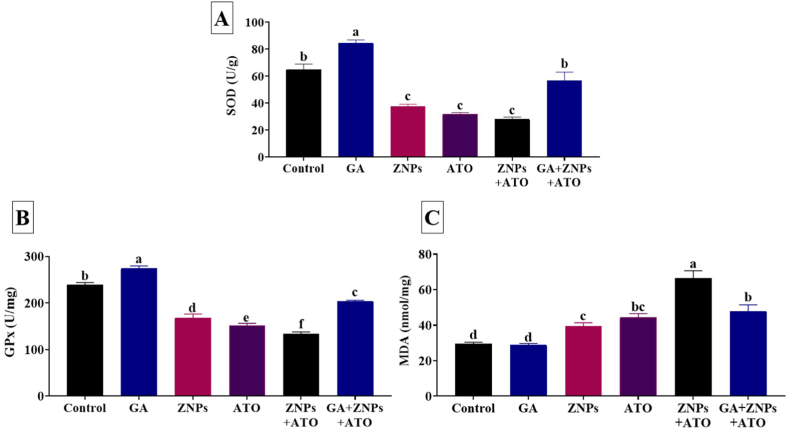
Effects of gallic acid (GA) on antioxidant enzymes including superoxide dismutase (SOD) (A) and glutathione peroxidase (GPx) (B) and the lipid peroxidation indicator, malondialdehyde (MDA) (C) in liver homogenate of rats exposed to zinc oxide nanoparticles (ZNPs) and/or arsenic trioxide (ATO) for 60 days. Data are expressed as the mean ± SE (n = 10). Columns carrying different superscripts are significantly different (one-way ANOVA) (*p* < 0.05).

In comparison with the control groups, ZNPs, ATO, and ZNPs+ATO groups showed a significant (*P* < 0.001) reduction in the hepatic SOD (58%, 49%, and 43%, respectively) (Fig. 1A) and GPx (70%, 63%, and 56%) activities (Fig. 1B). On the contrary, hepatic MDA concentration significantly (*P* < 0.001) increased in ZNPs, ATO, and ZNPs+ATO groups to 133%, 150%, and 224%, respectively, relative to the control group (Fig. 1C). Nonetheless, GA+ZNPs+ATO-treated rats showed a significant (*P* < 0.001) increases in hepatic GPx (152%) and SOD (203%) concentrations but a significant (*P* < 0.001) decrease in hepatic MDA level (72%) compared to the ZNPs+ATO exposed ones and the values returned to near-normal values.

### 3.5 Histopathological findings

**Fig. 2:**
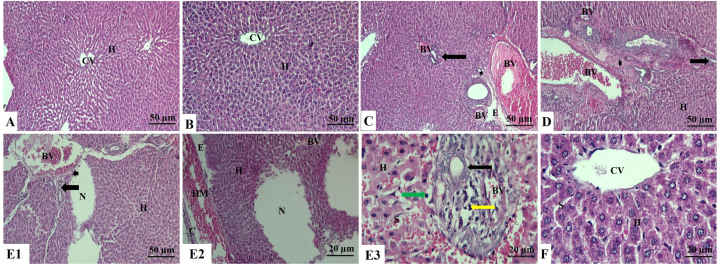
Liver sections of rat stained with H&E. **A:** Control group showing a normal architecture with normal hepatocytes in the hepatic cords and central vein. **B:** Gallic acid-treated (GA) group showing normal hepatocytes in the hepatic cords around the central vein. **C:** Zinc oxide nanoparticles-exposed (ZNPs) group showing a severely dilated, congested, and hypertrophied blood vessel wall, newly formed bile ductules, fibrotic portal area, and edema (H&E × 100). **D:** Arsenic trioxide-exposed (ATO) group showing hepatocytes disorganization, congested blood vessels, vasculitis, and hypertrophy of blood vessels wall associated with newly formed bile ductules and fibrosis in the portal area. **E1:** ZNPs+ATO co-exposed group showing disorganization of hepatocytes, necrotic areas along the hepatocytes, congested blood vessels, vasculitis and hypertrophy of blood vessels wall associated with newly formed bile ductules, and fibrosis in the portal area. **E2:** ZNPs+ATO co-exposed group showing hepatocytes disorganization, necrotic areas along the hepatocytes, congested blood vessels associated with prominent thickening and inflammatory cell infiltration of the liver capsule, subcapsular hemorrhage, and edema. **E3:** ZNPs+ATO co-exposed group showing disorganized hepatocytes, hepatocytes vacuolations, necrosis with pyknotic nuclei, congested blood vessels, sinusoids, vasculitis, newly formed bile ductules, mononuclear leucocytic infiltrations. **F:** ZNPs+ATO+GA-treated group showing normal hepatocytes in the hepatic cords around the central vein and sinusoids. **Abbreviations:** H: Hepatocytes, CV: Central vein, BV: Blood vessel, Arrow: Bile ductules, Arrowhead: Portal area, E: Edema, N: Necrotic area, C: Capsule, HM: Hemorrhage, Green arrow: Vacuolations, Yellow arrow: Mononuclear leucocytic infiltrations, CV: Central vein, S: Sinusoid. (For interpretation of the references to color in this figure legend, the reader is referred to the Web version of this article.)

As illustrated in Fig. 2 A and B, the untreated control and GA-treated groups liver sections showed normal histological features with normal hepatocytes without any necrosis or inflammation. Whilst ZNPs or ATO treatments induced hepatocyte disorganization and vacuolar degeneration with necrotic changes of the hepatocytes. Mononuclear cellular inflammatory infiltrations, mostly lymphocytes and macrophages, were also observed between hepatic cords. The central veins and blood sinusoids showed prominent dilation, congestion, and thickened wall of the central vein. There was perivascular edema and slight areas of hemorrhage with brown hemosiderin pigment deposition. In addition, the portal area revealed marked periportal fibrosis and hyperplastic bile duct epithelium associated with newly formed bile ductules (60 days). Prominent thickening of the liver capsule was associated with subcapsular congestion, hemorrhage, edema, and inflammatory cell infiltrations (Fig. 2C and D).

However, the ZNPs+ATO co-exposed group showed more severe hepatic damage than the groups individually exposed to each. The pathological alterations detected in the ZNPs+ATO co-exposed group were mainly widespread necrosis and vacuolar degeneration throughout the hepatic tissues, particularly the centrilobular cells (Fig. 2 E1, E2, and E3). On the contrary, GA treatment significantly reduced most histopathological perturbations, particularly inflammatory cell infiltration, and necrotic damage, in the hepatic tissues of ZNPs and ATO-co-exposed rats (Fig. 2F).

### 3.6 Histochemical findings

**Fig. 3:**
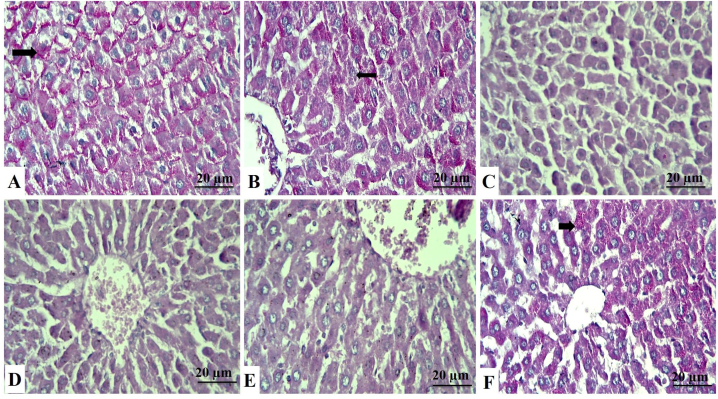
Liver sections stained with PAS, identified by their magenta color of glycogen granules in the cytoplasm of hepatocytes (arrows). **A:** Control group showing strong PAS reaction. **B:** Gallic acid-treated group showing strong positive cells. **C:** Zinc oxide nanoparticles-exposed (ZNPs) group showing wide areas that give negative results. **D:** Arsenic trioxide-exposed (ATO) group shows a decrease in cells' positivity to the stain. **E:** ZNPs+ATO co-exposed group showing wide areas of weak positivity to the stain. **F:** ZNPs+ATO+GA treated group showing an increase in the glycogen content of cells. (For interpretation of the references to color in this figure legend, the reader is referred to the Web version of this article.)

Additional liver sections from all groups were stained with PAS to gain more insight into the content of the vacuolar contents of hepatocytes (Fig. 3A–F). The PAS-stained hepatic tissue sections showed that exposure to ZNPs and/or ATO significantly diminished the hepatic contents of the glycogen granules compared to the control group. On the other hand, treatment with GA largely rescued the glycogen granules hepatic contents and regained the near-normal magenta color in the GA+ZNPs+ATO co-treated group.

### 3.7 Immunohistochemical evaluation

**Fig. 4:**
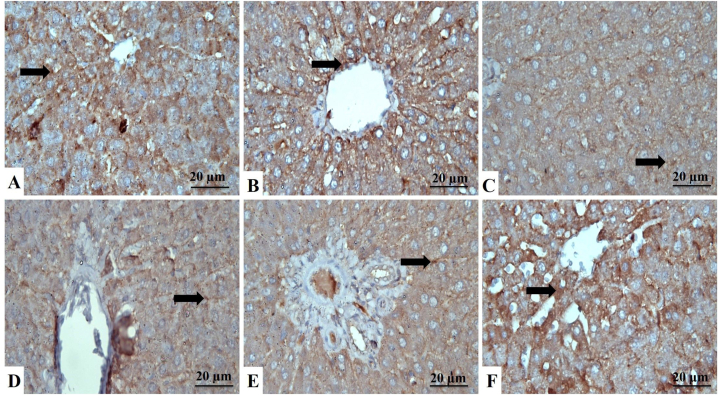
Liver sections stained with Bcl-2, identified by a brown cytoplasmic reaction to Bcl-2 antibodies in the hepatocytes (arrows). **A:** Control group showing strong immunoreactions. **B:** Gallic acid-treated group showing strong Bcl-2 antibodies mainly in the cytoplasm of hepatocytes. **C:** Zinc oxide nanoparticles-exposed (ZNPs) group showing the moderate reaction of hepatocytes. **D:** Arsenic trioxide-exposed (ATO) group showing moderate labeling cells. **E:** ZNPs+ATO co-exposed group showing weak expression of hepatocytes to the stain. **F:** ZNPs+ATO+GA treated group showing the increased positive reaction of hepatocytes to the stain. (For interpretation of the references to color in this figure legend, the reader is referred to the Web version of this article.)

**Fig. 5:**
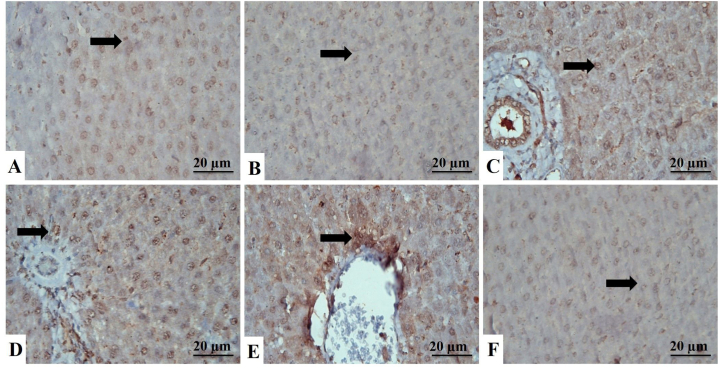
Liver sections stained with Bax, identified by brown cytoplasmic reaction in the hepatocytes (arrows). **A:** Control group showing weak immuno-labeling cells. **B:** Gallic acid-treated group showing weak immunoreaction of hepatocytes. **C:** Zinc oxide nanoparticles-exposed (ZNPs) group showing strong expression of hepatocytes to the stain. **D:** Arsenic trioxide-exposed (ATO) group shows hepatocytes' increased positive reaction to the stain. **E:** ZNPs+ATO co-exposed group showing strong immunoreactions. **F:** ZNPs+ATO+GA treated group showing the decreased reaction of cells. (For interpretation of the references to color in this figure legend, the reader is referred to the Web version of this article.)

The liver section of the control rats showed strong cytoplasmic reactions to the antiapoptotic protein Bcl-2 (Fig. 4A–F) and weak nuclear and cytoplasmic reactions of the proapoptotic protein Bax (Fig. 5A–F). Yet, as shown in Table 3, the ZNPs, ATO, and ZNPs+ATO-exposed rats showed a significantly (*P* < 0.001) decreased cytoplasmic immunoexpression of Bcl-2 (28%, 33%, and 23%, respectively) but increased nuclear and cytoplasmic immunoexpression of Bax (217%, 267%, and 236%, respectively) compared to the control group. Besides, the Bcl-2/Bax ratio significantly (*P* < 0.001) decreased in the livers of ZNPs, ATO, and ZNPs+ATO-exposed to be 13%, 12%, and 10%, respectively, compared to the control group. Nonetheless, the hepatic sections of GA+ZNPs+ATO-treated rats showed a significant (*P* < 0.001) increases in immunoexpression of Bcl-2 (351%) and Bcl-2/Bax ratio (933%) but a significant (*P* < 0.001) decreases in Bax (37%) compared to the ZNPs+ATO exposed ones.

**Table 3:** Effect of gallic acid (GA) oral dosing on hepatic Bcl2 and Bax immunoexpression and Bcl2/Bax ratio of rats exposed to zinc dioxide nanoparticles (ZNPs) and/or arsenic trioxide (ATO) for 60 days.

Hepatic immunoexpression	Control	GA	ZNPs	% of control*	ATO	% of control*	ZNPs+ATO	% of control*	GA+ZNPs+ATO	% of restoration^≠^
Bcl2	84.00^a^±2.04	80.33^a^±0.62	23.33^cd^ ± 1.03	28	28.00^c^±2.04	33	19.67^d^ ± 0.85	23	69.00^b^ ± 2.45	351
Bax	23.00^c^±1.63	19.33^c^±1.03	50.00^b^ ± 2.45	217	61.33^a^±1.55	267	54.33^b^ ± 2.05	236	20.33^c^±1.03	37
Bcl2/Bax ratio	3.70 ^ab^ ± 0.18	4.21^a^±0.25	0.47^c^±0.00	13	0.46^c^±0.05	12	0.37^c^±0.03	10	3.45^b^ ± 0.29	933

*% of control = (mean value of the intoxicated group/mean values of the control group)*100. ^≠^ % of restoration= (mean value of GA+ZNPs+ATO group/mean values of ZNPs+ATO group)*100. ND: Not detected. Means within the same row carrying different superscripts are significantly different at *p* < 0.05. The values shown are means ± SE. n = 10 group.

### 3.8 Zinc and arsenic hepatic residues

Relative to the control rats, significantly (*P* < 0.001) higher hepatic As content was recorded in rats exposed to ATO and ZNPs+ATO to be 172% and 205%, respectively (Table 4). On the contrary, the hepatic tissues of GA+ZNPs+ATO-treated rats had a significantly lower As content (59%) compared to the ZNPs+ATO-co-exposed ones.

**Table 4:** Effect of gallic acid (GA) oral dosing on liver content of arsenic (ATO) and zinc (Zn) of rats exposed to zinc dioxide nanoparticles (ZNPs) and/or arsenic trioxide (ATO) for 60 days.

Residues (ppm)	Control	GA	ZNPs	% of control*	ATO	% of control*	ZNPs+ATO	% of control*	GA+ZNPs+ATO	% of restoration^≠^
Arsenic (As)	0.39^c^±0.01	0.29 ^d^ ± 0.03	0.47^c^±0.02	121	0.67 ^b^ ± 0.03	172	0.80^a^±0.03	205	0.47^c^±0.02	59
Zinc (Zn)	24.63^c^±0.68	20.13^d^ ± 1.74	40.00^a^±0.82	162	32.43 ^b^ ± 1.05	132	41.23^a^±1.13	167	23.17 ^cd^ ± 0.78	56

*% of control = (mean value of the intoxicated group/mean values of the control group)*100. ≠ % of restoration= (mean value of GA + ZNPs + ATO group/mean values of ZNPs + ATO group)*100. ND: Not detected. Means within the same row carrying different superscripts are significantly different at p < 0.05. The values shown are means ± SE. n = 10 group.

Regarding Zn accumulation in the hepatic tissue, the ZNPs, ATO, and ZNPs+ATO exposed groups had significantly (*P* < 0.001) higher Zn content (162%, 132%, and 167%, respectively) compared to the control group. In contrast, the oral dosing of GA significantly (*P* < 0.001) reduced the ZNPs+ATO induced Zn accumulation in hepatic tissues to 56% compared to the ZNPs+ATO exposed ones, and the values returned near-normal values.

## 4 Discussion

The current study demonstrated that the ZNPs, alone or with ATO, significantly elevated the serum indicators of hepatocyte membrane disruption and cellular efflux [56]. Our findings were consistent with previous studies, which showed that ZNPs [10] and ATO [16,17] induced liver injury with severe oxidative stress. GA lessened ZNPs-ATO-induced hepatotoxicity by significant decreases in the elevated AST, ALT, and ALP levels, with the protection of hepatocytes' efficiency. These findings were similar to those previously reported by Zhu et al. [31], who found that GA significantly decreases serum hepatic enzymes and normalizes the expression of pro-inflammatory cytokines, interleukins, and COX2.

Our results showed obvious hyperlipidemia in the ZNPs and/or ATO-exposed rats. Similar findings were obtained by Jiang et al. [57], who confirmed that ATO exposure elevated the lipid profiles and induced hyperlipidemia in rats. Additionally, Mondal et al. [58] found that adult male rats orally administered 3 mg ATO/kg b. wt for 30 consecutive days showed a marked increase in serum TC, TG, and LDL but decreased HDL levels. Moreover, Moatamed et al. [59] recorded significant increases in TG, total cholesterol, and LDL serum levels with an extensive decrease in serum HDL in the ZNPs-exposed group, and the toxicity was dose-dependent. GA significantly normalized the total cholesterol, TG, LDL, and VLDL and increased HDL concentrations in diabetic rats [60]. Interestingly, the same authors reported that GA improved lipid profiles, oxidative stress, and inflammation by regulating microRNA expressions associated with endothelial dysfunction. Of note, the results of LDL-C and VLDL-C reported herein should be considered in light of one limitation. Despite the several earlier studies that depended on Friedewald et al. [47] equation in estimating LDL-C and VLDL-C [[61], [62], [63]], as in our case. Yet, the equation's use of a fixed factor of 5 to represent the link between TG and VLDL-C makes it susceptible to inaccuracy at low LDL-C and/or high TG levels, where errors in calculating VLDL-C are accentuated [64,65]. Hence, to reduce the possibility of error and improve the reliability of LDL-C readings, direct LDL-C assays could be used in future studies.

Our findings illustrated that the direct bilirubin (conjugated hyperbilirubinemia) level was significantly elevated in ZNPs, either alone or with ATO indicating hepatic or prehepatic jaundice. However, the rise in conjugated bilirubin suggests cholestasis or hepatocellular injury [66]. Total bilirubin elevation can occur in cholestatic or hepatocellular disorders, and elevated serum-conjugated bilirubin suggests hepatocellular disease or biliary obstruction [67]. The catabolic product, bilirubin, is produced when the catalase and peroxidase enzymes act on the heme moieties in the hemoglobin structure. Subsequently, the total hepatic bilirubin is converted to conjugated bilirubin by the glucuronate pathway [68]. The histopathological findings support the earlier results as various pathological changes were recorded in the hepatic tissues of the ATO and/or ZNPs-exposed rats. Moreover, the ZNPs + ATO-co-exposed rats showed more severe histopathological alterations than those exposed to each individually.

Herein, the hepatic tissue of ZNPs and/or ATO-exposed rats showed a noticeable reduction of the antioxidant enzyme activities (SOD and GPx) and increased lipid peroxidative damage. Several endogenous antioxidative indicators, such as SOD and GPx, are activated to counteract synthesized free radicals and protect cells from oxidative injury [69]. When the liberated ROS free radicals exceed the cell's antioxidant capacity to scavenge them, oxidative stress arises from the peroxidation of membrane lipids [70]. Several studies have previously recorded oxidative stress following ZNPs and/or ATO exposure [10,15,17]. However, GA co-administration protects the liver against adverse changes in oxidative stress and increases the reduced levels of endogenous antioxidant biomarkers [71]. GA suppressed *tert*-butyl hydroperoxide (*t*-BHP)-induced hepatic cytotoxicity and protected the tissues against ROS oxidation [72]. The potential antioxidant of GA may be attributed to the restoration of cellular antioxidants [73], mitochondrial enzymatic activities (including citrate synthase and cytochrome *c* oxidase), and intracellular ATP levels [74].

Our biochemical results were confirmed by the histological and immunohistochemical findings (Fig. 2, Fig. 3, Fig. 4, Fig. 5 and Table 3). Histopathologically, different changes indicate the hepatotoxic effects of ZNPs or ATO exposure, including vacuolar degeneration with necrotic changes of the hepatocytes. The hepatic tissues of ZNPs or ATO-treated rats showed mononuclear cellular inflammatory infiltrations, mostly lymphocytes and macrophages between hepatic cords. Perivascular edema and hemorrhage indicate the interaction between toxicants and the interstitial hepatic tissues, causing a variety of inflammatory responses [75]. Similar results were reported by Zhang et al. [76] and Almansour et al. [77], who established that sinusoidal dilatation, lobular and portal triads inflammatory cells infiltration, necrosis, hydropic degeneration, hepatocytes apoptosis is found in ZNPs and ATO treated rats, respectively.

In the current study, ZNPs and/or ATO-mediated cell death is associated with apoptosis, as evidenced by the recorded significant decline in Bcl-2 and elevation in Bax immunoreactivities. Besides, the Bcl2/Bax ratio was significantly reduced in the hepatic tissues of ZNPs and/or ATO- exposed rats. The Bcl2 to Bax ratio is an important determinant of apoptotic cell death [78,79]. In this regard, Sharma et al. [80] proposed that oxidative stress might lead to apoptosis of liver cells during *in vivo* exposure to ZNPs. Besides, ATO-induced ROS production has been reported to be responsible for initiating apoptotic consequences in liver cells [81]. On the other hand, GA treatment significantly upset the ZNPs and ATO-induced apoptotic events in the hepatocytes. Similarly, GA considerably suppressed the apoptotic changes in liver cells resulting from ZNPs and ATO exposure [82]. The defensive effect of GA against oxidative stress might contribute to the antiapoptotic effect of GA [82,83].

Significant accumulation of As or Zn was evident in the liver of rats individually exposed to ATO and/or ZNPs. Comparably, Sharma et al. [80] reported that sub-acute oral exposure to ZNPs resulted in a significant accumulation of NPs in the liver of mice. Additionally, Dubey et al. [84] verified the strong correlation between As accumulation in the hepatic tissues in ATO-intoxicated rats and the resultant hepatic damage. NPs are well-known for their huge surface area, allowing heavy metals to adsorb [85]. Heavy metals can enter the organism as free ions or *N*P-heavy metal complexes, and NPs can carry their movement inside the organism [86]. It has also been reported that high-activity ZNPs penetrate their target tissues and form a thick coating of NPs that cannot be absorbed by phagocytes and enter the lymph flow with rapid systemic distribution [87]. The earlier facts could explain the significant hepatic accumulation of As in the ZNPs+ATO co-exposed rats than those exposed to ATO alone. On the contrary, GA treatment significantly reduced the As and Zn accumulation in the hepatic tissues of ZNPs and ATO-co-exposed rats. This effect could be highly linked to the GA chelating activity [88]. Deguchi [89] evaluated the GA and Zn interaction by potentiometric titration and verified the complexation between them, signifying that Zn prefers bonding to the GA carbonyl group.

## 5 Conclusion

Our results established the protective efficacy of co-administration of GA against hepatotoxicity in adult male rats caused by subchronic ZNPs and ATO exposure. GA normalized biochemical, histological, and oxidative stress profiles and minimized the co-toxic effects associated with its effective antioxidant properties. Therefore, it will be appropriate to recommend using GA as an adjuvant remedy to NPs and potential heavy metal pollutants.

## Author contribution statement

Khaled Abo-EL-Sooud: yasmina Abd El Hakim: Conceived and designed the experiments; Performed the experiments; Analyzed and interpreted the data; Contributed reagents, materials, analysis tools or data; Wrote the paper.

Mohamed Hashem: Conceived and designed the experiments; Performed the experiments; Contributed reagents, materials, analysis tools or data.

Abeer El-Metwally: Conceived and designed the experiments; Analyzed and interpreted the data; Contributed reagents, materials, analysis tools or data; Wrote the paper.

Bayan Hassan: Conceived and designed the experiments; Analyzed and interpreted the data; Contributed reagents, materials, analysis tools or data.

Hayat El-Nour: Analyzed and interpreted the data; Contributed reagents, materials, analysis tools or data.

## Funding statement

This research was funded by 10.13039/501100002386Cairo University in a project entitled “Assessment of the risk hazards of co-exposure to nanomaterials and environmental contaminants with mitigation strategies using natural products” (Cairo university projects-12-2021).

## Data availability statement

Data will be made available on request.

## Declaration of competing interest

The authors declare that they have no known competing financial interests or personal relationships that could have appeared to influence the work reported in this paper.
